# Hexagonal-Boron-Nitride-Reinforced Butyl/Chloroprene Rubber Composites for Tire Curing Bladder Applications

**DOI:** 10.3390/polym18091112

**Published:** 2026-04-30

**Authors:** Baran Cetin, Mehmet Durmus Calisir, Ali Kilic, Islam Shyha

**Affiliations:** 1 Department of Polymer Science and Technology, Istanbul Technical University, 34369 Istanbul, Türkiye; cetinb21@itu.edu.tr; 2 Brisa Bridgestone Sabanci Tyre Manufacturing and Trading Inc., 41310 Kocaeli, Türkiye; 3 TEMAG Labs, Faculty of Textile Technologies and Design, Istanbul Technical University, 34437 Istanbul, Türkiye; mehmetdurmus.calisir@erdogan.edu.tr (M.D.C.); alikilic@itu.edu.tr (A.K.); 4 Faculty of Engineering and Architecture, Recep Tayyip Erdogan University, 53100 Rize, Türkiye; 5 Department of Industrial Engineering, College of Engineering, University of Business and Technology, Jeddah 21448, Saudi Arabia; 6 School of Computing Engineering and the Built Environment, Edinburgh Napier University, Edinburgh EH10 5DT, UK

**Keywords:** hexagonal boron nitride, butyl rubber, thermal conductivity, rheology, fatigue resistance, viscoelasticity, tire curing bladder

## Abstract

This study investigates a thermal management strategy for butyl/chloroprene rubber (IIR/CR) bladder compounds by incorporating hexagonal boron nitride (h-BN) as a thermally conductive filler to enhance heat transfer efficiency. Compounds containing 0, 10, 25, and 33 wt% h-BN were prepared via solution mixing to ensure uniform dispersion and subsequently vulcanized using a hot press. The materials were characterized in terms of morphology, cure behavior using a moving die rheometer (MDR), thermal conductivity, crosslink density, mechanical properties, and dynamic mechanical analysis (DMA). The incorporation of h-BN significantly enhanced thermal performance, nearly doubling the thermal conductivity at 33 wt%. MDR measurements demonstrated that this improved heat transfer capability accelerated the thermal onset of vulcanization, effectively reducing scorch time. Mechanical testing revealed a systematic increase in stiffness at application-relevant low strain levels (25–50%), attributed to hydrodynamic reinforcement, accompanied by a progressive increase in elongation at break. This enhanced extensibility is associated with the presence of lamellar h-BN platelets, which facilitate stress redistribution and promote dynamic chain mobility under deformation. DMA showed that h-BN incorporation increased the storage modulus and intensified the Payne effect, confirming the formation of a robust physical filler network. Overall, the incorporation of h-BN delivers a formulation pathway for energy-efficient tire curing bladders by significantly improving heat transfer efficiency and dimensional stability.

## 1. Introduction

Heat-conductive polymer composites have gained increasing attention in transportation, electronics, and energy systems because they combine the processing advantages of polymers with improved heat dissipation under service conditions [1,2,3]. However, most polymers and especially elastomers are inherently poor thermal conductors (∼0.16–0.22 W m^−1^ K^−1^) due to weak phonon transport along disordered molecular chains and significant interfacial scattering. This limitation becomes critical in rubber applications where rapid and uniform heat transfer is required, such as tire curing bladders and other thermally cycled components. The prevailing strategy to overcome low conductivity is to integrate thermally conductive fillers that form continuous heat transfer pathways while preserving the elastomeric response of the base polymer [1,2,3,4].

Various ceramic (SiC, Al_2_O_3_, AlN, ZnO, BN) and carbon-based fillers (carbon black, graphite, carbon fibers, carbon nanotubes, reduced graphene oxide) have been studied for this purpose [5,6,7,8,9,10,11,12,13,14]. Among them, high aspect ratio morphologies (whiskers, platelets, nanoplatelets) are particularly effective because they can reach the percolation threshold at lower volume fractions, allowing formation of conductive networks with less severe penalties to processability and toughness. In addition, advanced filler architectures including three-dimensional (3D) networks, hierarchical assemblies, and hybrids combining fillers of different dimensionalities can further minimize interfacial thermal resistance, which often limits composite performance. However, the gains in conductivity reported in the literature frequently rely on high filler loadings; while these loadings facilitate network formation, they can depress crosslink density, raise viscosity, and deteriorate mechanical compliance trade-offs that are unacceptable for many rubber applications [15,16,17,18]. Among the available thermally conductive fillers, hexagonal boron nitride (h-BN) is distinguished by its combination of high intrinsic thermal conductivity (≈250–300 W m^−1^ K^−1^ along the basal plane) and excellent electrical insulation [19,20]. Its layered, graphite-like structure allows it to act as a heat conductor and an electrical insulator simultaneously, making it highly suitable for thermally conductive and electrically insulating composites.

h-BN has been widely employed as a functional filler in diverse polymer matrices, with most studies emphasizing how filler loading governs the resulting thermal and related properties of the composites [21]. In solution styrene–butadiene rubber, comparative studies clarified the distinct roles of graphite and h-BN in building thermomechanical networks. Fan and Cho reported that, at fixed loading, graphite provides stronger mechanical reinforcement due to formation of a stronger filler network and higher carbon rubber interfacial reinforcement, while hybrid systems show monotonic increases in strength with increasing graphite fraction. Intriguingly, thermal conductivity in the hybrid can slightly trail that of all graphite analogues yet remain comparable to those of all BN cases at equal loading [22].

In silicone matrices, electroneutral nitride ceramics with high intrinsic κ offer a complementary design space. Co-filling addition-cure liquid silicone rubber (ALSR) with AlN and BN (fixed 8:2 mass ratio) increases κ up to ~3.4× relative to neat ALSR, while modestly increasing dielectric constant and loss. Additionally, slight advancement at the onset of mass loss and higher high-temperature residue collectively indicate a small net gain in thermal stability at higher filler loadings. Moreover, changes in volume resistivity before and after thermal aging highlight how filler content and thermal history jointly affect charge transport and dielectric reliability, which are critical for high-voltage insulation applications [23].

Beyond steady state heat transfer, filler chemistry and interfacial interactions play a key role in determining radiation tolerance and crosslink behavior in aggressive environments. In fluororubber (FKM), nanoscale h-BN not only boosts κ but also strengthens the composite through specific interactions (e.g., B–F bonding signatures by XPS/FT-IR), increases cross link density, and markedly improves radiation stability. At 10 parts per hundred rubber (phr) h-BN, tensile strength and modulus increase compared with neat FKM, and the absorbed dose required to halve elongation at break increases ~1.7×. These improvements are attributed to restricted oxygen diffusion and reduced radiation-induced oxidation. These findings indicate that thermally conductive fillers can provide multifunctional benefits by improving heat transfer, mechanical reinforcement, and radiation resistance when their dispersion and interfacial interactions are well controlled [24].

Classical studies on boron nitride (BN)-filled silicone rubbers further describe the processing–property space. Zhou et al. reported that thermal conductivity increases monotonically with BN content up to approximately 60 vol%, although very high loadings negatively affect mechanical performance and processability; around 40 vol% therefore represents a practical balance. Particle size exerts a pronounced influence: at a fixed filler volume fraction, larger BN particles yield higher thermal conductivity consistent with longer phonon mean free paths but can reduce strength relative to finer grades. Conversely, careful bimodal or multimodal size distributions densify packing and promote percolative pathways, elevating thermal conductivity without causing catastrophic embrittlement [25].

Tire curing bladders represent a particularly demanding and industrially critical application where improved thermal transport is directly convertible to energy savings and higher productivity. During vulcanization, the bladder delivers pressure and heat to the green tire; low bladder conductivity slows through-thickness heat flow, extends cure times, and exacerbates temperature gradients that can lead to non-uniform crosslinking [26,27]. Butyl rubber (IIR) is the incumbent matrix for bladders by virtue of its low gas permeability, chemical resistance, and thermal stability features that are further enhanced when phenolic curing systems are applied. Traditional bladder compounds incorporate carbon black, plasticizers (e.g., castor oil), and resin cure packages; despite their maturity, they still suffer from the intrinsic thermal transport limitations of rubber. Designing IIR-based compounds with significantly improved thermal conductivity without sacrificing extensibility, fatigue resistance, and manufacturability would therefore address a long-standing bottleneck in tire production [28,29,30,31,32,33].

The present study investigates h-BN-filled butyl/chloroprene (IIR/CR) rubber compounds tailored for tire curing bladder applications. Thermally conductive fillers such as BN, AlN, Al_2_O_3_, graphite, and carbon-based materials have been widely examined in polymer composites; however, most of these studies have predominantly focused on epoxy, silicone rubber, fluororubber, or general-purpose elastomer matrices. In contrast, IIR-rich bladder compounds incorporating a fixed minor fraction of CR and a phenolic resin curing system have received limited attention [1,27], despite their direct industrial relevance in tire manufacturing. Accordingly, this work addresses this gap by providing a systematic and application-oriented evaluation of h-BN-reinforced IIR/CR compounds within a realistic bladder formulation framework. Unlike previous studies that primarily emphasize thermal conductivity, the present study adopts a holistic approach by examining the coupled effects of h-BN on heat transfer behavior, cure characteristics, crosslink development, filler network formation, mechanical response, and crack growth resistance. Through the combined analysis of thermal conductivity, MDR response, swelling-derived crosslink density, tensile properties, DMA behavior, and morphology, this work defines the processing–structure–property relationships governing h-BN-reinforced IIR/CR composites. The results offer a formulation and characterization framework for the design of thermally efficient and mechanically durable tire curing bladder compounds, with potential implications for reducing curing time and energy consumption.

## 2. Experimental

### 2.1. Materials

h-BN with a purity of 99.85%, an average particle size of 65–75 nm, and a true density of 2.3 g/cm^3^ was supplied from Nanografi Nanotechnology, Ankara, Türkiye. Zinc oxide was employed with a heat loss of 0.2% and a purity of 99.85% used as a curing activator. Phenolic butyl resin was characterized by a softening point of 84 °C and a methylol content of 8.32% and served as the curing resin. Stearic acid, with a melting point of 54 °C, was employed as a processing aid. Carbon black (N234 grade) with a specific surface area of 112.6 m^2^/g was used as a reinforcing filler. Chloroprene rubber, exhibiting a Mooney viscosity of 47 MU, was incorporated at a minor level and served as a halogen-containing rubber, acting as a halogen donor/activator to facilitate phenolic resin curing and improve interfacial interactions within the rubber matrix, while butyl rubber showed a Mooney viscosity ML (1 + 8) at 125 °C of 53 MU and an unsaturation level of 1.68 mol%.

### 2.2. Compound Preparation

Rubber compounds containing 0%, 10%, 25%, and 33 wt% of h-BN were prepared using a 10 wt% toluene solution. In all formulations, the rubber matrix was intentionally designed as a 95/5 phr blend of butyl rubber (IIR) and chloroprene rubber (CR), corresponding to a total rubber content of 100 phr. The CR used in this study was a commercial mercaptan-grade 2-chloro-1,3-butadiene polymer with medium crystallization tendency and was supplied in talc-coated chip form. It exhibited a nominal Mooney viscosity of ML (1 + 4) at 100 °C of 47 MU. The CR content was selected as 5 phr to introduce a limited halogen-containing co-rubber phase capable of acting as a halogen donor/activator for the phenolic resin curing system, while maintaining the IIR-rich matrix required for tire curing bladder applications. Since the CR content was kept constant in all formulations, its contribution was considered part of the fixed base curing system, and the comparative evaluation focused on the effect of h-BN loading rather than the independent curing behavior of pure CR. All other ingredients were added relative to this rubber basis, following standard rubber compounding conventions. The formulation details are summarized in Table 1.

The mixtures were mechanically stirred at room temperature at 2000 rpm for 24 h to ensure uniform dispersion of h-BN within the rubber matrix. After mixing, the solvent was removed by evaporation, followed by drying in a vacuum oven to ensure complete removal of residual toluene. The obtained rubber compounds were then further homogenized using a two-roll mill at room temperature. All compounds were cured in an LPC029 hot press (Fontijne, Niles, MI, USA) at 170 °C for 75 min. Subsequently, test specimens were prepared, and their mechanical, dynamic mechanical, and thermal conductivity properties were analyzed.

### 2.3. Characterization Methods

#### 2.3.1. Thermogravimetric Analysis

Thermogravimetric analysis (TGA) was conducted using a TGA Q500 instrument (TA Instruments, New Castle, DE, USA) over a temperature range of 30 to 800 °C, at a heating rate of 20 °C/min under a nitrogen atmosphere with a gas flow of 20 mL/min.

#### 2.3.2. Morphological Characterization

Morphological observations were carried out using an Olympus SZH light microscope (Olympus Corporation, Tokyo, Japan) equipped with an Analysis Particle Inspector. In addition, scanning electron microscopy (SEM) analysis was performed using a Vega Tescan instrument (TESCAN, Brno, Czech Republic).

#### 2.3.3. Rheological Analysis

Cure characteristics were evaluated using a moving die rheometer (MDR 2000, Alpha Technologies, Hudson, OH, USA) under isothermal conditions at 171 °C for 75 min, in accordance with ASTM D5289 [34]. The rheometer operated at a frequency of 1.667 Hz and a temperature range of 25–200 °C. The cure rate index (CRI), which quantifies the rate of the curing reaction, was calculated using Equation (1):

(1) $$CRI=\frac{100}{t_{90}-t_{s2}}[\%]$$

where t_90_ is the optimum cure time and t_s2_ is the scorch time [35].

#### 2.3.4. Crosslink Density Measurement

Equilibrium swelling experiments were carried out in accordance with ISO 1817 [36] to estimate the crosslink density (CLD) of the vulcanized compounds. Circular specimens (17.8 mm in diameter and 2 mm in thickness) were cut from each vulcanizate and weighed prior to swelling ($m_{0}$). The specimens were then immersed in toluene at room temperature for 72 h. After swelling, the samples were removed, gently blotted with filter paper to remove surface solvent, and weighed immediately ($m_{1}$). The swollen specimens were subsequently dried in an oven at 70 °C until a constant mass was obtained ($m_{2}$).

(2) $$M_{c}=\frac{-\rho_{p}V_{s}V_{r}^{\frac{1}{3}}}{ln\left(1-V_{r}\right)+V_{r}+\chi V_{r}^{2}}$$

(3) $$V_{r}=\frac{\frac{m_{2}-m_{f}}{\rho_{p}}}{\frac{m_{2}-m_{f}}{\rho_{p}}+\frac{m_{1}-m_{2}}{\rho_{s}}}$$

(4) $$V_{c}=\frac{1}{{2M}_{c}}$$

The crosslink density ($V_{c}$) was calculated using the Flory–Rehner approach (Equations (2)–(4)) [35], where $M_{c}$ is the average molecular weight between crosslinks, $V_{r}$ is the volume fraction of rubber in the swollen network, and $V_{s}$ is the molar volume of toluene (106.4 cm^3^/mol). The densities of the solvent and the rubber blend were denoted as $\rho_{s}$ and $\rho_{p}$, respectively. The effect of fillers was accounted for by including the filler mass term ($m_{f}$), defined as the total mass of carbon black and h-BN, as specified in the corresponding equations.

(5) $$\chi =\chi_{\beta }+\frac{V_{s}({\delta_{s}-\delta_{r})}^{2}}{RT}$$

The Flory–Huggins polymer–solvent interaction parameter (χ) used in Equation (2) was calculated using Equation (5) [35], where the entropy contribution $\chi_{\beta }$ was taken as 0.34, $\delta_{s}$ is the solubility parameter of toluene (18.35 (J/cm^3^)^1/2^), R is the universal gas constant (8.314 J·mol^−1^·K^−1^), and T is the absolute temperature (293.15 K). The solubility parameter of the rubber blend ($\delta_{r}$) was estimated by a volume-fraction mixing rule (Equation (6)) [35] using the constituent rubbers, butyl rubber (IIR) and chloroprene rubber (CR), with their respective solubility parameters and volume fractions ($\phi_{\mathrm{IIR}}$ and $\phi_{\mathrm{CR}}$).

(6) $$\delta_{r}=\delta_{IIR}\phi_{IIR}+\delta_{CR}\phi_{CR}$$

#### 2.3.5. Mechanical Testing

Each mechanical test was conducted a minimum of five times, and the reported values represent the average results. Tensile tests were performed using a Zwick/Roell universal testing machine (Zwick/Roell, Ulm, Germany) at a crosshead speed of 500 mm/min under ambient conditions (23 ± 2 °C), in accordance with ASTM D412 [37] using Type C dumbbell specimens. The results include tensile strength, elongation at break, and modulus values at selected elongation levels.

#### 2.3.6. Dynamic Mechanical Analysis and Crack Growth Testing

Dynamic mechanical analysis (DMA) was performed using a GABO Eplexor 500 N instrument (Netzsch, Selb, Germany). Temperature sweep tests were conducted in tension mode from −40 °C to 180 °C, with a heating rate of 3 °C/min, at a frequency of 52 Hz and a constant strain of 0.2%. To assess filler–filler interactions, the Payne effect was evaluated using a DMA (Netzsch, Selb, Germany) in tension mode. Strain sweep tests were conducted at 100 °C across a strain amplitude range of 0.1% to 5%. The magnitude of the Payne effect (ΔE′) was quantified as the difference between the storage modulus at low strain (0.1%) and high strain (5%), calculated using Equation (7):

(7) $$\Delta E^{\prime }=E_{low}^{\prime }-E_{high}^{\prime }$$

Crack growth behavior was evaluated using a De Mattia flexing tester, which is designed to assess the resistance of vulcanized rubber specimens to crack initiation and propagation under repeated flexural stress. Specially molded samples were subjected to cyclic flexing at a frequency of 300 rpm and a controlled temperature of 95 ± 2 °C over a specified duration. The extent of flex cracking and subsequent crack growth was recorded to determine the material’s durability under dynamic mechanical fatigue.

#### 2.3.7. Thermal Conductivity Measurement

Thermal conductivity measurements of the vulcanized rubber compounds were performed using a DTC 300 guarded hot plate apparatus (TA Instruments, New Castle, DE, USA), in accordance with ASTM D5470 [38] and based on the guarded heat flow method outlined in ASTM E1530 [39]. The device comprises two temperature-controlled metal plates between which the test specimen is placed. Following the application of a specified pressure and temperature, the instrument measures thermal resistance across the sample. Through-plane thermal conductivity was evaluated at 25 °C under a pressure of 25 psi. Measurements were conducted on cylindrical pad-shaped specimens with a diameter of 5 cm and a thickness of 2 mm.

## 3. Results and Discussion

### 3.1. Thermal Stability of Compounds

**Figure 1:**
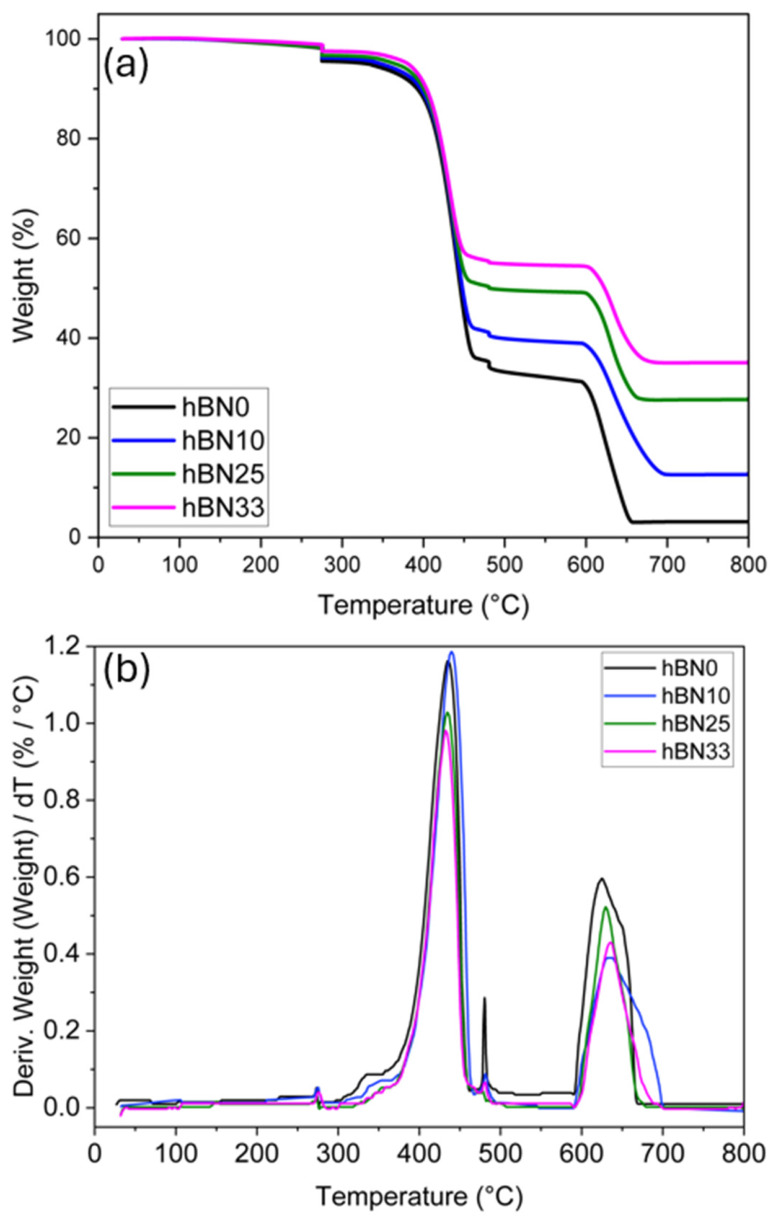
TGA (**a**) and DTG (**b**) curves of h-BN0, h-BN10, h-BN25 and h-BN33 samples.

The thermal degradation behavior of the neat and h-BN-reinforced IIR/CR composites was systematically investigated by thermogravimetric analysis (TGA) and derivative thermogravimetry (DTG) under a nitrogen atmosphere, so that the observed trends represent intrinsic thermal behavior rather than oxidative degradation. As shown in Figure 1 and summarized in Table 2, incorporating h-BN leads to a pronounced increase in the onset of thermal decomposition. The temperature at 5% weight loss (T_5_) increases from 331.2 °C for the unfilled reference to 381.1 °C for the composite containing 33 wt% h-BN. This increase is consistent with reduced early-stage mass loss in the filled systems and can be rationalized by the decreased mass fraction of volatile constituents at higher h-BN loadings under constant total batch mass and the presence of platelet like h-BN, which may hinder the transport of volatile degradation products during the initial decomposition stage [23].

The DTG curves reveal a two-step degradation mechanism for all formulations. The first maximum degradation temperature (T_1max_), associated with the primary decomposition of the rubber matrix, shows a modest shift toward lower temperatures with increasing h-BN content, decreasing from 442.1 °C (h-BN0) to 432.2 °C (h-BN33) [40]. This shift should be interpreted only as a change in the temperature of the maximum degradation rate peak (T_1max_), while the onset of degradation is governed by T_5_.

At elevated temperatures, the h-BN-filled composites exhibit improved resistance to high-temperature mass loss. The second DTG peak (T_2max_), attributed to the decomposition of the carbonaceous phase, shifts from 621.1 °C for the unfilled compound to 634.5 °C for the 33 wt% h-BN composites. Consistent with this trend, the residual mass at 800 °C increases markedly from 3.11% to 35.29% with increasing h-BN content, indicating a substantially higher char/residue yield in the highly filled systems. Overall, the combined increases in T_5_, T_2max_, and residual mass demonstrate enhanced thermal stability at high temperatures with increasing h-BN loading, while the modest shift in T_1max_ remains limited to the position of the maximum degradation rate peak.

### 3.2. Rheological Properties

In moving die rheometer (MDR) testing, the torque–time trace characterizes the curing behavior of the compounds at the test temperature. Before vulcanization, thermal softening drives the torque to a baseline; this minimum torque (ML) reflects the initial viscosity/stiffness of the uncured compound. As crosslinking proceeds, torque rises toward a plateau; the maximum torque (MH) is widely used as a proxy for final stiffness and, indirectly, effective crosslink density. The cure extent (ΔS = MH − ML) describes the overall network development, though in filled systems it combines both chemical crosslinking and the physical contribution of the filler network. Scorch times (ts_1_ and ts_2_) mark when torque first exceeds ML by 1 and 2 dN·m and indicate the onset of curing while characteristic cure times t_10_, t_50_, and t_90_ denote 10%, 50%, and 90% of completion of the total torque rise, respectively. The CRI, computed as in Equation (1), provides a compact measure of cure kinetics, with higher values indicating faster overall cure.

**Figure 2:**
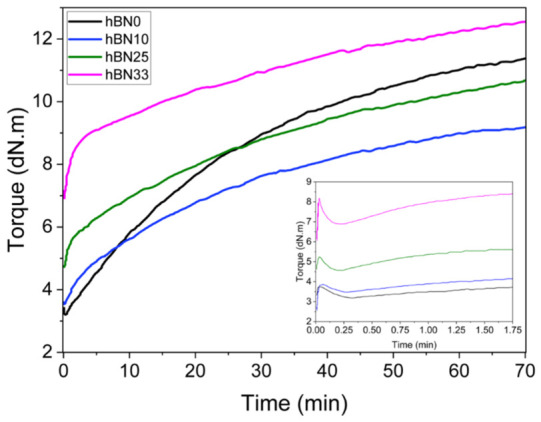
MDR Graphs of h-BN0, h-BN10, h-BN25 and h-BN33 compounds.

Figure 2 and Table 3 summarize the curing characteristics of the compounds. The addition of h-BN led to a systematic increase in minimum torque (ML) from 3.19 dN·m for h-BN0 to 6.88 dN·m for h-BN33. This rise reflects increased initial viscosity, consistent with the development of a stronger physical filler network that hinders matrix flow [35]. This interpretation is also supported by the monotonic increase in Payne effect presented in Section 3.6 dynamic properties (E′_0_: 9.04 → 24.90 MPa, ΔE′: 4.59 → 20.88 MPa).

Regarding cure kinetics, h-BN exerts a dual effect by accelerating the thermal onset while limiting the reaction rate. Early-stage parameters (ts_1_, t_10_) decreased significantly with filler loading; for instance, ts_1_ dropped from 3.71 min for h-BN0 to 0.92 min for h-BN33. This accelerated onset may be associated with the high thermal conductivity of h-BN, which minimizes thermal lag and speeds up heat transfer. In contrast, the optimum cure time t_90_ remains essentially unchanged and CRI decreases at the highest loading (2.13 to 1.90 min^−1^), suggesting that the late-stage completion of cure is not accelerated and may be affected by increased viscosity and restricted mobility at high h-BN contents.

The maximum torque (MH) exhibited non-monotonic behavior, reflecting competing mechanisms. Initially, MH decreased from 11.37 dN·m (h-BN0) to 9.27 dN·m (h-BN10), which may be associated with a reduced development of an elastically effective chemical network and/or interfacial slip facilitated by the platelet-like h-BN. This interpretation is consistent with the increased elongation at break observed in tensile test results provided in section 3.5 [41]. At higher loadings, the recovery of MH should not be interpreted solely as an increase in chemical crosslink density; rather, rigid h-BN platelets contribute to the torque response through hydrodynamic reinforcement, where the increased filler volume fraction reduces the deformable rubber fraction and amplifies the local strain in the surrounding matrix. Together with the strengthened filler network, as indicated by the monotonic increase in the Payne effect, this physical reinforcement contribution becomes more influential and causes MH to recover and surpass h-BN0, reaching 12.57 dN·m for h-BN33 [42]. It should be noted that the torque values reflect not only chemical crosslink formation but also the increasing total filler content and filler-induced physical network formation, since the total phr of the compound increases with h-BN loading.

Importantly, the cure extent ΔS (MH–ML) decreased from 8.18 ± 0.05 dN·m (h-BN0) to 5.69 dN·m for the h-BN-filled compounds (Table 3), indicating a reduced cure extent. This trend is consistent with the crosslink density results discussed in Section 3.3, supporting a suppressed development of the elastically effective chemical network with increasing h-BN content, even though the absolute torque level is elevated due to filler-related physical contributions.

### 3.3. Crosslink Density

The CLD is a key structural parameter governing the final performance of vulcanized rubber compounds. To assess the influence of h-BN on network formation, equilibrium swelling experiments were conducted and the effective CLD values were calculated using the Flory–Rehner approach. As summarized in Table 4, the swelling-based CLD decreases markedly with increasing h-BN loading, from 4.50 × 10^−4^ (h-BN0) to 3.33 × 10^−4^ (h-BN10), 2.04 × 10^−4^ (h-BN25), and 9.63 × 10^−5^ (h-BN33). It should be noted that, in filled systems, swelling-derived CLD may also be influenced by filler–rubber interactions and constrained swelling; nevertheless, the pronounced reduction indicates a lower development of an elastically effective network at higher h-BN contents under the applied curing conditions.

These findings are broadly consistent with the MDR results, where the cure extent was evaluated using ΔS (MH–ML) (Table 3). Compared to h-BN0 (8.18 ± 0.05 dN·m), all h-BN-containing compounds exhibit lower ΔS values (5.80 ± 0.06, 6.09 ± 0.04, and 5.69 ± 0.05 dN·m for h-BN10, h-BN25, and h-BN33, respectively), indicating reduced torque development during vulcanization. Since MDR torque reflects the combined contribution of chemical crosslinking and filler-related physical effects, the slight non-monotonic difference between h-BN10 and h-BN25 may originate from dispersion and filler network contributions.

### 3.4. Thermal Conductivity

**Figure 3:**
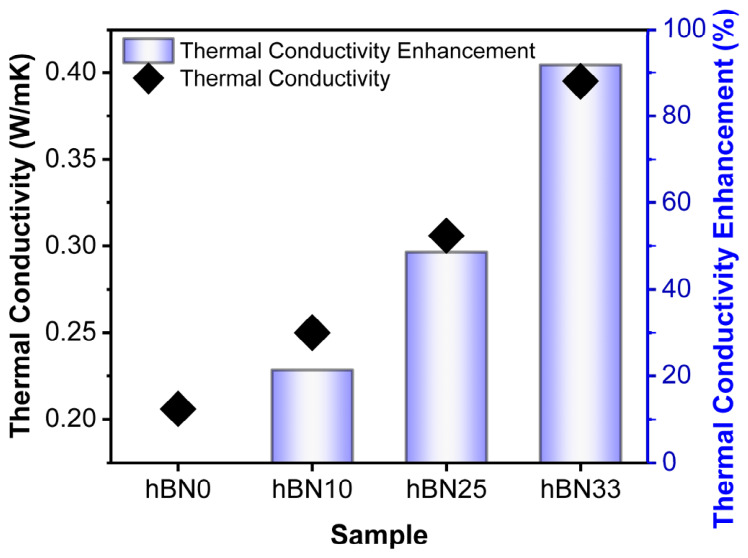
Thermal conductivity and enhancement percentage of h-BN0, h-BN10, h-BN25 and h-BN33 samples.

As presented in Figure 3, the thermal conductivity of the h-BN/rubber composites increases monotonically with filler loading, rising from 0.206 W·m^−1^·K^−1^ for h-BN0 to 0.395 W·m^−1^·K^−1^ for h-BN33, corresponding to a total enhancement of approximately 92%. At lower loadings (≤10 wt%), the improvement is moderate, as heat transfer is primarily limited by the high interfacial thermal resistance between the randomly dispersed, isolated h-BN particles and the rubber matrix. This resistance includes thermal contact resistance due to imperfect filler–matrix coupling and thermal boundary (Kapitza) resistance arising from phonon mismatch between the constituent phases. Since the h-BN used in this study was not surface-modified, the interfacial resistance may further limit phonon transport, particularly at low filler contents where conductive pathways are not sufficiently developed [43].

However, a pronounced increase is observed at 33 wt% loading. This enhancement is attributed to the higher h-BN content, which increases the probability of platelet–platelet proximity and local heat transfer pathway formation within the rubber matrix, as commonly reported for BN-filled polymer composites [44]. This interpretation is also supported by the SEM observations given in Section 3.7 where higher h-BN loadings resulted in rougher, more layered fracture surfaces and locally concentrated filler-rich domains. These morphological features suggest that increasing h-BN content promotes more effective local thermal pathways, although interfacial thermal resistance between the filler and rubber matrix remains an important limiting factor.

### 3.5. Mechanical Properties

**Figure 4:**
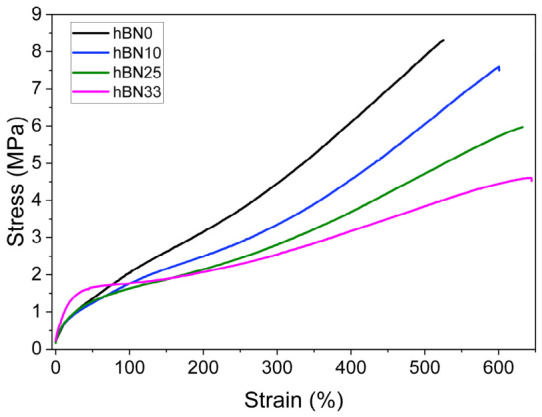
Stress–strain curves of h-BN-filled rubber composites.

Uniaxial tensile properties were evaluated, and the moduli at fixed strains (M25, M50, M100, M200, and M300) were defined as the engineering stress values at 25%, 50%, 100%, 200%, and 300% strain, respectively. Ultimate tensile strength (UTS) and elongation at break were also recorded (Table 4), and representative stress–strain curves are shown in Figure 4. At low strains, the modulus remains essentially unchanged up to 25 wt% h-BN (M25: 0.96 ± 0.03 MPa for h-BN0, 0.93 ± 0.03 MPa for h-BN10, and 0.97 ± 0.04 MPa for h-BN25), followed by a clear increase for h-BN33 (1.41 ± 0.12 MPa). A similar trend is observed for M50 (1.36 ± 0.05 → 1.24 ± 0.04 → 1.29 ± 0.05 → 1.66 ± 0.10 MPa). In contrast, the moduli at higher strains decrease progressively with increasing h-BN content (e.g., M300: 4.45 ± 0.20 MPa for h-BN0 vs. 3.35 ± 0.03, 2.80 ± 0.06, and 2.55 ± 0.18 MPa for h-BN10, h-BN25, and h-BN33, respectively), indicating that the h-BN platelets do not provide effective reinforcement under large deformations. This behavior can be attributed to the limited load transfer efficiency of platelet-type h-BN fillers. The lamellar structure of h-BN promotes interfacial slippage and facilitates chain mobility under large deformation, thereby reducing the effective reinforcement capability of the filler. Given that tire curing bladders typically operate under moderate dimensional strains (30–40%), the low-strain moduli (M25–M50) are the most application-relevant tensile indicators for dimensional stability.

As shown in Figure 4 and summarized in Table 5, UTS decreases monotonically with h-BN loading from 8.31 ± 0.06 MPa (h-BN0) to 7.60 ± 0.42 MPa (h-BN10), 5.97 ± 0.24 MPa (h-BN25), and 4.61 ± 0.10 MPa (h-BN33). Conversely, elongation at break increases from 525 ± 18% to 601 ± 20%, 632 ± 19%, and 642 ± 25% with increasing h-BN content. Taken together with the reduction in high-strain moduli, this combination of lower strength and higher extensibility is consistent with a non-reinforcing filler response in tension: h-BN increases stiffness mainly at small strains (most pronounced at 33 wt%) but does not enhance the load-bearing capacity at large deformations. This is consistent with the measured Payne effect (ΔE′), which indicates a pronounced filler network contribution at low strain that weakens under increasing deformation, aligning with the reduced high-strain tensile moduli [41].

### 3.6. Dynamic Mechanical and Crack Growth Properties

**Figure 5:**
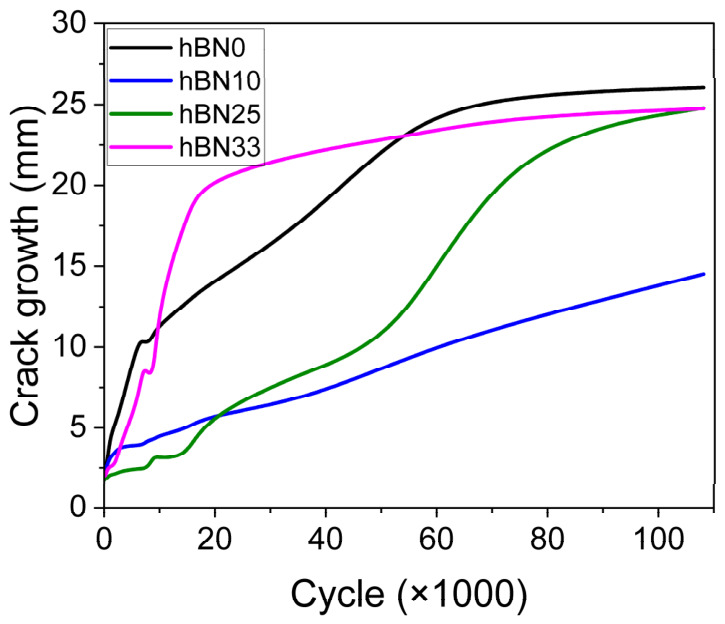
Crack growth performance of h-BN0, h-BN10, h-BN25 and h-BN33 compounds.

Figure 5 presents the crack growth behavior of the rubber compounds under cyclic loading. The unfilled sample (h-BN0) exhibited the fastest crack propagation, indicating the lowest fatigue resistance. Incorporation of h-BN notably improved crack growth resistance, though the effect varied with filler content. Among the composites, the 25 wt% h-BN sample (h-BN25) showed the best fatigue performance up to approximately 15,000 cycles, maintaining the lowest crack growth rate among all formulations. The 10 wt% h-BN compound (h-BN10) also exhibited reduced crack propagation compared with the unfilled rubber but showed a steadier, moderate increase across cycles. At higher loadings, crack growth accelerated after the initial phase. The 33 wt% h-BN sample (h-BN33) initially exhibited limited crack extension but experienced a sharp rise after about 6000 cycles, likely due to filler agglomeration and interfacial debonding, suggesting that excessive filler content may reduce structural integrity.

**Figure 6:**
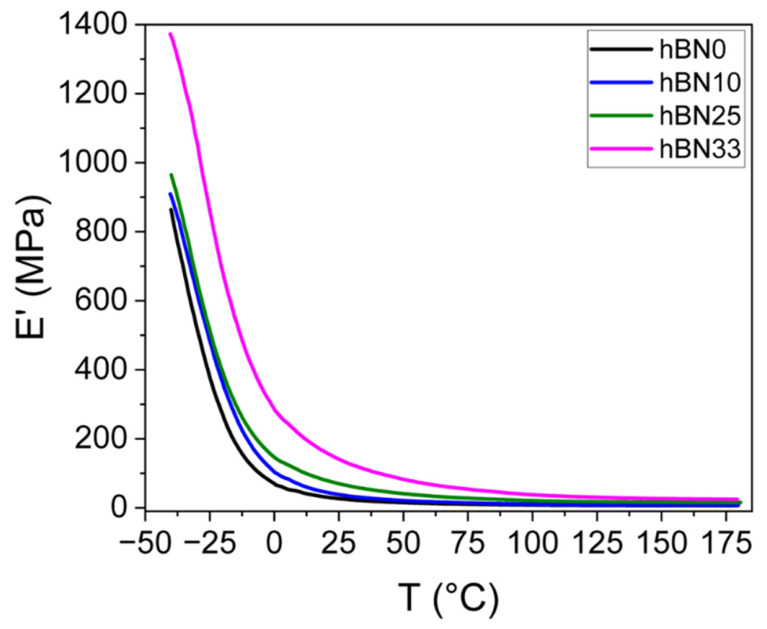
Storage modulus as a function of temperature for h-BN0, h-BN10, h-BN25 and h-BN33 compounds.

As shown in Figure 6, the incorporation of h-BN provides a consistent stiffness enhancement compared to the h-BN0 compound over the entire temperature range. The storage modulus (E′) increases with h-BN content over the entire temperature range, indicating that reinforcement is not limited to a specific thermal region. The modulus difference is modest at room temperature but becomes more evident at higher temperatures. At 25 °C, E′ increases from 26.35 MPa (h-BN0) to 139.99 MPa (h-BN33). Importantly, the reinforcement remains evident at elevated temperature: at 150 °C, the 33 wt% compound still exhibits 26.57 MPa compared to 6.54 MPa for h-BN0.

All formulations soften with temperature; based on the 80–150 °C interval, the normalized retention E′ (150 °C)/E′ (80 °C) is 0.651 (h-BN0) and 0.535 (h-BN33), indicating that the absolute modulus remains much higher with h-BN, although the high-loading compound shows a somewhat larger drop over this interval. Overall, the E′–T trends are consistent with filler-driven reinforcement, which maintains higher stiffness at service-relevant temperatures. Practically, higher E′ at elevated temperature implies improved resistance to deformation under load for elastomeric components.

**Figure 7:**
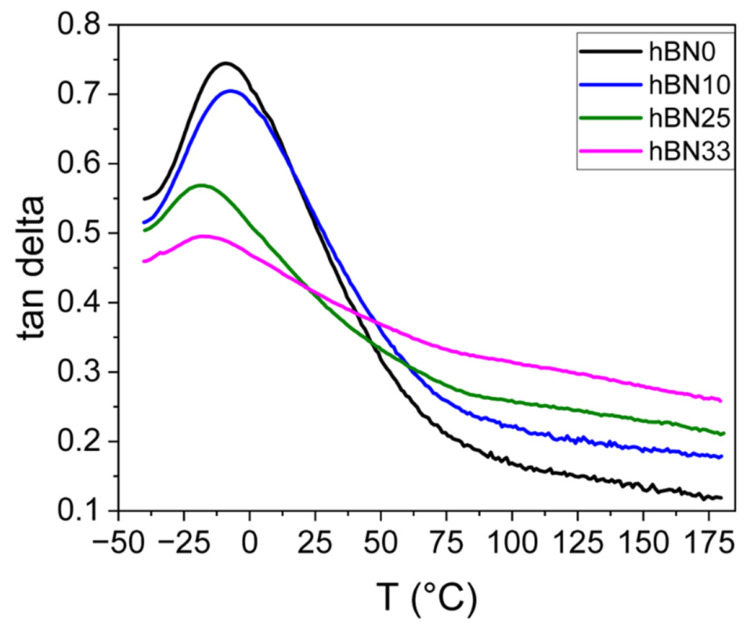
Loss factor (tan δ) as a function of temperature for h-BN0, h-BN10, h-BN25 and h-BN33 compounds.

Figure 7 shows the temperature dependence of tanδ. The tanδ peak in the glass-transition region (α-relaxation) decreases in magnitude with increasing h-BN content, indicating reduced damping near Tg and a more elastic response, consistent with the higher E′ values. The position of the α-relaxation peak exhibits no pronounced shift with increasing h-BN content. In the ambient to moderate temperature range (approximately 0–50 °C), the h-BN-filled compounds generally show lower tanδ than the reference, suggesting reduced hysteretic losses within this interval. At higher temperatures, tanδ becomes comparatively higher for the highly filled compound, which may reflect additional dissipation associated with filler network and interfacial contributions, consistent with the observed increase in Payne effect.

**Figure 8:**
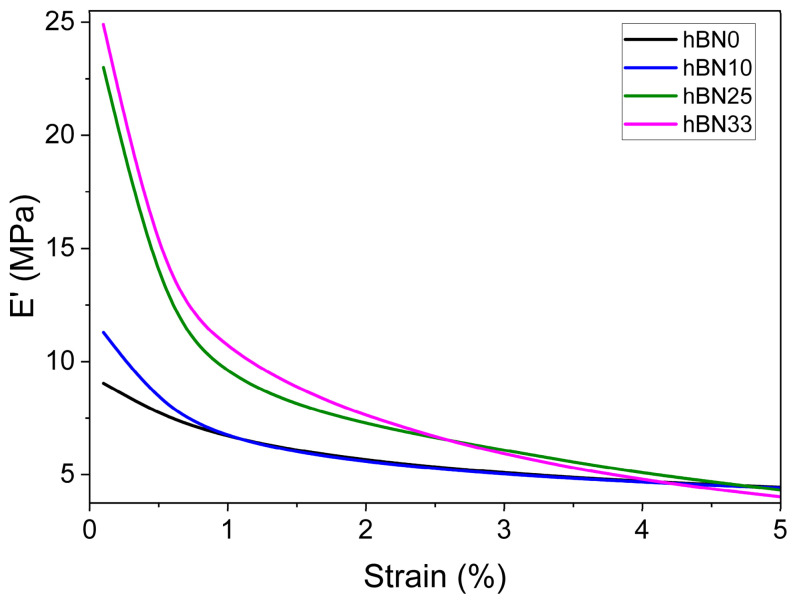
Strain sweep of h-BN0, h-BN10, h-BN25 and h-BN33 compounds at 100 °C.

Figure 8 and Table 6 show strain sweep DMA results in tension at 52 Hz and 100 °C over a dynamic strain range of 0.1–5%. All h-BN-filled compounds exhibit a pronounced Payne effect, characterized by a continuous decrease in storage modulus (E′) with increasing strain due to the breakdown of filler–filler interactions and partial collapse of the filler network. Both the initial storage modulus (E′_0_) and the magnitude of the Payne effect (ΔE′) increase systematically with h-BN content, indicating the progressive formation of a rigid filler network [45].

The modulus drop is more pronounced for h-BN25 and h-BN33, indicating a more strain-sensitive filler network dominated by filler–filler interactions. In contrast, h-BN10 exhibits a more gradual decline in E′, consistent with lower filler loading and a more homogeneous dispersion state. Overall, these findings support the formation of a percolated filler network at higher h-BN contents, in agreement with the microstructural observations obtained from SEM analysis.

### 3.7. Morphological Properties

**Figure 9:**
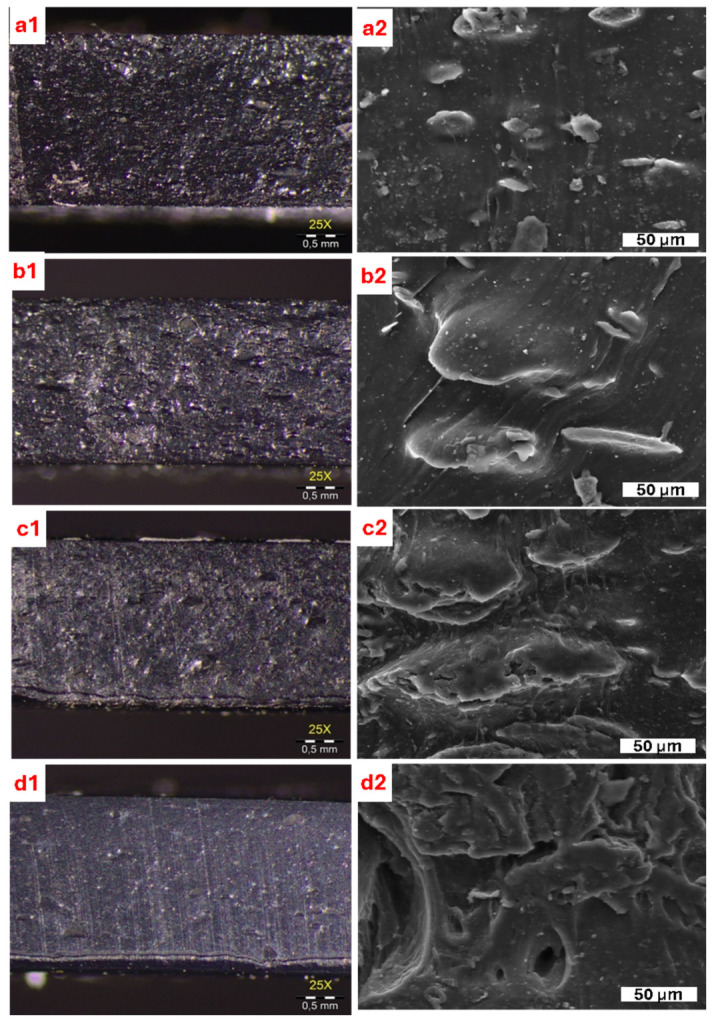
Optical microscopy (**left**, 25×) and SEM micrographs (**right**, scale bar: 50 µm) of IIR/CR bladder compounds containing (**a1**,**a2**) 0 wt% h-BN, (**b1**,**b2**) 10 wt% h-BN, (**c1**,**c2**) 25 wt% h-BN, and (**d1**,**d2**) 33 wt% h-BN.

As shown in Figure 9, optical micrographs indicate a macroscopically uniform appearance across the cross-sections, with no evidence of large (mm scale) agglomeration across all compositions. SEM images reveal a progressive change in fracture surface morphology with increasing h-BN loading, characterized by a rougher, more layered topography and the appearance of micron-scale filler-rich domains at 10–33 wt%. These observations indicate increasing local heterogeneity while maintaining overall uniform dispersion. This hierarchical morphology plays a key role in enabling thermally conductive pathway formation at higher h-BN contents.

## 4. Conclusions

In this study, hexagonal boron nitride (h-BN) was incorporated into IIR/CR rubber blends as a functional filler to address the low thermal conductivity of tire curing bladders, and its effect on the compound properties was investigated compared to the control. The addition of h-BN significantly increased the thermal conductivity of the compounds from 0.206 W·m^−1^·K^−1^ for h-BN0 to 0.395 W·m^−1^·K^−1^ for h-BN33, reaching a maximum enhancement of approximately 92% at 33 wt% loading. This improvement was attributed to the increased h-BN content and the formation of local heat transfer pathways within the rubber matrix.

Regarding curing characteristics, the inclusion of h-BN accelerated the onset of vulcanization due to enhanced heat transfer, reducing the scorch time significantly. Simultaneously, the maximum torque increased, confirming the physical reinforcement provided by the rigid filler particles. Mechanical properties, especially the low-strain moduli relevant to bladder service, were improved at high h-BN loading. M25 increased from 0.96 to 1.41 MPa and M50 from 1.36 to 1.66 MPa for h-BN33, indicating enhanced dimensional stability under moderate deformation. In contrast, tensile strength decreased from 8.31 to 4.61 MPa, while elongation at break increased from 525% to 642%, suggesting reduced stress-transfer efficiency at large deformation. Dynamic mechanical analysis further confirmed the reinforcing effect of h-BN, as the storage modulus at 25 °C increased from 26.35 to 139.99 MPa and the Payne effect increased from 4.59 to 20.88 MPa. In addition, the crack growth results indicated that intermediate h-BN loading, particularly 25 wt%, provided a more favorable balance in dynamic durability. From all these aspects, it can be concluded that h-BN serves as a multifunctional filler that offers an optimal balance of thermal efficiency, processability, and mechanical performance for high-performance tire curing bladders.

## Figures and Tables

**Table 1:** Rubber compound formulations.

Ingredients (phr)	h-BN0	h-BN10	h-BN25	h-BN33
Butyl rubber	95	95	95	95
h-BN	0	19	59	85
Carbon black	50	50	50	50
Polychloroprene rubber	5	5	5	5
Castor oil	5	5	5	5
Stearic acid	1	1	1	1
Zinc oxide	5	5	5	5
Phenolic butyl resin	10	10	10	10
TOTAL	171	190	230	256

**Table 2:** Characteristic data of h-BN0, h-BN10, h-BN25, h-BN33 TGA and DTG curves.

Sample	T_5_ (°C)	T_1max_ (°C)	T_2max_ (°C)	Residual wt% (800 °C)
h-BN0	331.22	442.06	621.07	3.11
h-BN10	347.96	439.60	632.31	12.86
h-BN25	365.50	435.97	630.05	27.89
h-BN33	381.05	432.24	634.46	35.29

**Table 3:** MDR results of rubber compounds with different h-BN loadings.

	Unit	h-BN0	h-BN10	h-BN25	h-BN33
ML	dN·m	3.19 ± 0.03	3.47 ± 0.02	4.57 ± 0.02	6.88 ± 0.04
MH	dN·m	11.37 ± 0.08	9.27 ± 0.09	10.66 ± 0.09	12.57 ± 0.07
ΔS (MH-ML)	dN·m	8.18 ± 0.05	5.80 ± 0.06	6.09 ± 0.04	5.69 ± 0.05
CRI	%/min	2.13 ± 0.02	2.19 ± 0.02	2.02 ± 0.04	1.90 ± 0.01
Ts1	min	3.71 ± 0.12	2.96 ± 0.10	1.46 ± 0.09	0.92 ± 0.05
Ts2	min	7.88 ± 0.2	9.20 ± 0.21	7.55 ± 0.18	3.68 ± 0.14
t10	min	2.99 ± 0.05	1.42 ± 0.03	0.75 ± 0.03	0.59 ± 0.01
t50	min	18.77 ± 0.14	16.86 ± 0.16	17.43 ± 0.15	12.56 ± 0.22
t90	min	54.76 ± 0.22	54.84 ± 0.24	57.07 ± 0.18	56.35 ± 0.24
S′@Ts2	dN·m	5.19 ± 0.06	5.47 ± 0.02	6.57 ± 0.02	8.88 ± 0.03
S′@t10	dN·m	4.01 ± 0.02	4.04 ± 0.02	5.18 ± 0.01	7.45 ± 0.04
S′@t50	dN·m	7.28 ± 0.09	6.35 ± 0.07	7.63 ± 0.09	9.73 ± 0.13
S′@t90	dN·m	10.55 ± 0.23	8.68 ± 0.05	10.04 ± 0.06	12.02 ± 0.11

**Table 4:** Crosslink density test results.

	Unit	h-BN0	h-BN10	h-BN25	h-BN33
$V_{c}$ (10^−5^)	mol/g	45	33.3	20.4	9.63

**Table 5:** Ultimate tensile strength (UTS), % elongation and modulus values of rubber samples.

	Unit	h-BN0	h-BN10	h-BN25	h-BN33
M25	MPa	0.96 ± 0.03	0.93 ± 0.03	0.97 ± 0.04	1.41 ± 0.12
M50	MPa	1.36 ± 0.05	1.24 ± 0.04	1.29 ± 0.05	1.66 ± 0.10
M100	MPa	2.05 ± 0.07	1.76 ± 0.04	1.63 ± 0.06	1.77 ± 0.10
M200	MPa	3.15 ± 0.10	2.49 ± 0.04	2.14 ± 0.06	2.07 ± 0.13
M300	MPa	4.45 ± 0.20	3.35 ± 0.03	2.80 ± 0.06	2.55 ± 0.18
UTS	MPa	8.31 ± 0.06	7.60 ± 0.42	5.97 ± 0.24	4.61 ± 0.10
Elongation at break	%	525.38 ± 17.98	600.54 ± 20.24	632.31 ± 19.49	642.27 ± 24.65

**Table 6:** Initial modulus and Payne effect of the compounds.

	h-BN0	h-BN10	h-BN25	h-BN33
E′_0_ (MPa)	9.04	11.29	23.00	24.90
ΔE′ (MPa)	4.59	6.87	18.67	20.88

## Data Availability

The original contributions presented in this study are included in the article. Further inquiries can be directed to the corresponding author.

## References

[1] Shiva M., Dallakeh M.K., Ahmadi M., Lakhi M. (2021). Effects of Silicon Carbide as a Heat Conductive Filler in Butyl Rubber for Bladder Tire Curing Applications. Mater. Today Commun..

[2] Guo Y., Ruan K., Shi X., Yang X., Gu J. (2020). Factors Affecting Thermal Conductivities of the Polymers and Polymer Composites: A Review. Compos. Sci. Technol..

[3] Niu H., Ren Y., Guo H., Małycha K., Orzechowski K., Bai S.-L. (2020). Recent Progress on Thermally Conductive and Electrical Insulating Rubber Composites: Design, Processing and Applications. Compos. Commun..

[4] Wang Z.-H., Lu Y.-L., Liu J., Dang Z.-M., Zhang L.-Q., Wang W. (2011). Preparation of Nanoalumina/EPDM Composites with Good Performance in Thermal Conductivity and Mechanical Properties. Polym. Adv. Technol..

[5] Zhang Y., Fan Y., Kamran U., Park S.-J. (2022). Improved Thermal Conductivity and Mechanical Property of Mercapto Group-Activated Boron Nitride/Elastomer Composites for Thermal Management. Compos. Part A Appl. Sci. Manuf..

[6] Cecen V., Thomann R., Mülhaupt R., Friedrich C. (2017). Thermal Conductivity, Morphology and Mechanical Properties for Thermally Reduced Graphite Oxide-Filled Ethylene-Vinyl Acetate Copolymers. Polymer.

[7] Eksik O., Bartolucci S.F., Gupta T., Fard H., Borca-Tasciuc T., Koratkar N. (2016). A Novel Approach to Enhance the Thermal Conductivity of Epoxy Nanocomposites Using Graphene Core–Shell Additives. Carbon.

[8] Guo H., Li X., Li B., Wang J., Wang S. (2017). Thermal Conductivity of Graphene/Poly(Vinylidene Fluoride) Nanocomposite Membrane. Mater. Des..

[9] Li Q., Guo Y., Li W., Qiu S., Zhu C., Wei X., Chen M., Liu C., Liao S., Gong Y. (2014). Ultrahigh Thermal Conductivity of Assembled Aligned Multilayer Graphene/Epoxy Composite. Chem. Mater..

[10] Yang X., Zhu J., Yang D., Zhang J., Guo Y., Zhong X., Kong J., Gu J. (2020). High-efficiency improvement of thermal conductivities for epoxy composites from synthesized liquid crystal epoxy followed by doping BN fillers. Compos. Part B Eng..

[11] Yang S.-Y., Ma C.-C.M., Teng C.-C., Huang Y.-W., Liao S.-H., Huang Y.-L., Tien H.-W., Lee T.-M., Chiou K.-C. (2010). Effect of Functionalized Carbon Nanotubes on the Thermal Conductivity of Epoxy Composites. Carbon.

[12] Zhang X.-X., Meng Q.-J., Wang X.-C., Bai S.-H. (2011). Poly(adipic acid-hexamethylene diamine)-Functionalized Multi-Walled Carbon Nanotube Nanocomposites. J. Mater. Sci..

[13] Cui X., Ding P., Zhuang N., Shi L., Song N., Tang S. (2015). Thermal Conductive and Mechanical Properties of Polymeric Composites Based on Solution-Exfoliated Boron Nitride and Graphene Nanosheets: A Morphology-Promoted Synergistic Effect. ACS Appl. Mater. Interfaces.

[14] Hong J.-P., Yoon S.-W., Hwang T., Oh J.-S., Hong S.-C., Lee Y., Nam J.-D. (2012). High Thermal Conductivity Epoxy Composites with Bimodal Distribution of Aluminum Nitride and Boron Nitride Fillers. Thermochim. Acta.

[15] Fang L., Wu W., Huang X., He J., Jiang P. (2015). Hydrangea-like Zinc Oxide Superstructures for Ferroelectric Polymer Composites with High Thermal Conductivity and High Dielectric Constant. Compos. Sci. Technol..

[16] Chen W., Wang Z., Zhi C., Zhang W. (2016). High Thermal Conductivity and Temperature Probing of Copper Nanowire/Upconversion Nanoparticles/Epoxy Composite. Compos. Sci. Technol..

[17] Gu J., Yang X., Lv Z., Li N., Liang C., Zhang Q. (2016). Functionalized Graphite Nanoplatelets/Epoxy Resin Nanocomposites with High Thermal Conductivity. Int. J. Heat Mass Transf..

[18] Kim H.S., Kim J.H., Kim W.Y., Lee H.S., Kim S.Y., Khil M.-S. (2017). Volume Control of Expanded Graphite Based on Inductively Coupled Plasma and Enhanced Thermal Conductivity of Epoxy Composite by Formation of the Filler Network. Carbon.

[19] Xiao Y.-J., Wang W.-Y., Lin T., Chen X.-J., Zhang Y.-T., Yang J.-H., Wang Y., Zhou Z.-W. (2016). Largely Enhanced Thermal Conductivity and High Dielectric Constant of Poly(vinylidene fluoride)/Boron Nitride Composites Achieved by Adding a Few Carbon Nanotubes. J. Phys. Chem. C.

[20] Lin Z., Mcnamara A., Liu Y., Moon K.-S., Wong C.-P. (2014). Exfoliated Hexagonal Boron Nitride-Based Polymer Nanocomposite with Enhanced Thermal Conductivity for Electronic Encapsulation. Compos. Sci. Technol..

[21] Wang L., Qin Z., Huang L., Lin X., Wang Z., Li Z., Li Z., Lu S. (2025). Modified Boron Nitride Reinforced Natural Rubber Composites and Molecular Dynamics Simulation. Polym. Compos..

[22] Fan Y., Cho U.R. (2019). Effects of Graphite and Boron Nitride Based Fillers on Mechanical, Thermal Conductive, and Thermo-Physical Properties in Solution Styrene–Butadiene Rubber. Polym. Compos..

[23] Ou Z., Gao F., Zhao H., Dang S., Zhu L. (2019). Research on the Thermal Conductivity and Dielectric Properties of AlN and BN Co-Filled Addition-Cure Liquid Silicone Rubber Composites. RSC Adv..

[24] Wu Z., Shen H., Wang Y., Jiao L., Chen Y., Gong X., Lin M. (2024). Enhancing Mechanical Property, Thermal Conductivity, and Radiation Stability of Fluorine Rubber through Incorporation of Hexagonal Boron Nitride Nanosheets. Polym. Compos..

[25] Zhou W., Qi S., Zhao H., Liu N. (2007). Thermally Conductive Silicone Rubber Reinforced with Boron Nitride Particle. Polym. Compos..

[26] Deniz V., Karaagac B., Ceyhan N. (2007). Thermal Stability of Butyl/EPDM/Neoprene Based Rubber Compounds. J. Appl. Polym. Sci..

[27] Shiva M., Ahmadi M., Esmaili E., Zivdar M. (2024). Heat Diffusivity and Mechanical Properties of a Tire Bladder Composite in the Presence of Ceramic Fillers. Polym. Bull..

[28] Ivan G., Bugaru E., Volintiru T. (1988). A New Activation System for Resin Curing of Butyl Rubber. Acta Polym..

[29] Samadi A., Kashani M.R. (2010). Effects of Organo-Clay Modifier on Physical–Mechanical Properties of Butyl-Based Rubber Nano-Composites. J. Appl. Polym. Sci..

[30] Miranian Z., Movahed S.O., Movahed N.O. (2024). The Phenolic Cured Butyl Rubber Reclamation Using Different De-Crosslinking Agents. Prog. Rubber Plast. Recycl. Technol..

[31] Khavarnia M., Movahed S.O. (2016). Butyl Rubber Reclamation by Combined Microwave Radiation and Chemical Reagents. J. Appl. Polym. Sci..

[32] Caron P.A., Larreteguy A.E., Porta P.F. (2017). Cure Kinetics of Butyl Rubber Cured by Phenol Formaldehyde Resin. Lat. Am. Appl. Res. Int. J..

[33] Nair C. (2004). Advances in Addition-Cure Phenolic Resins. Prog. Polym. Sci..

[34] (2019). Standard Test Method for Rubber Property—Vulcanization Using Rotorless Cure Meters.

[35] Bingöl C.B., Polat Ş., Atapek Ş.H. (2024). Utilization of Composite ZnO in SBR/BR Compounds and Its Effect on the Cure and Physicomechanical Properties. Express Polym. Lett..

[36] (2024). Rubber, Vulcanized or Thermoplastic—Determination of the Effect of Liquids.

[37] (2021). Standard Test Methods for Vulcanized Rubber and Thermoplastic Elastomers—Tension.

[38] (2024). Standard Test Method for Thermal Transmission Properties of Thermally Conductive Electrical Insulation Materials.

[39] (2025). Standard Test Method for Evaluating the Resistance to Thermal Transmission by the Guarded Heat Flow Meter Technique.

[40] Zhi Y.-R., Yu B., Yuen A.C.Y., Liang J., Wang L.-Q., Yang W., Lu H.-D., Yeoh G.-H. (2018). Surface Manipulation of Thermal-Exfoliated Hexagonal Boron Nitride with Polyaniline for Improving Thermal Stability and Fire Safety Performance of Polymeric Materials. ACS Omega.

[41] Panda J.N., Bijwe J., Pandey R.K. (2019). Role of Micro and Nano-Particles of h-BN as a Secondary Solid Lubricant for Improving Tribo-Potential of PAEK Composite. Tribol. Int..

[42] Heinrich G., Klüppel M., Vilgis T.A. (2002). Reinforcement of Elastomers. Curr. Opin. Solid State Mater. Sci..

[43] Moradi S., Calventus Y., Román F., Ruiz P., Hutchinson J.M. (2020). Epoxy composites filled with boron nitride: Cure kinetics and the effect of particle shape on the thermal conductivity. J. Therm. Anal. Calorim..

[44] Kim Y.-K., Chung J.-Y., Lee J.-G., Baek Y.-K., Shin P.-W. (2017). Synergistic Effect of Spherical Al_2_O_3_ Particles and BN Nanoplates on the Thermal Transport Properties of Polymer Composites. Compos. Part A Appl. Sci. Manuf..

[45] Cöcen H., Kızılcan N. (2026). Hybrid filler and coupling agent: Effect of partial replacement of carbon black with talc and silane on properties of natural rubber compound. Express Polym. Lett..

